# Tumor necrosis factor (TNF)-receptor 1 and 2 mediate homeostatic synaptic plasticity of denervated mouse dentate granule cells

**DOI:** 10.1038/srep12726

**Published:** 2015-08-06

**Authors:** Denise Becker, Thomas Deller, Andreas Vlachos

**Affiliations:** 1 Institute of Clinical Neuroanatomy, Neuroscience Center, Goethe-University Frankfurt, Frankfurt D-60590, Germany

## Abstract

Neurological diseases are often accompanied by neuronal cell death and subsequent deafferentation of connected brain regions. To study functional changes after denervation we generated entorhino-hippocampal slice cultures, transected the entorhinal pathway, and denervated dentate granule cells *in vitro*. Our previous work revealed that partially denervated neurons respond to the loss of input with a compensatory, i.e., homeostatic, increase in their excitatory synaptic strength. TNFα maintains this denervation-induced homeostatic strengthening of excitatory synapses. Here, we used pharmacological approaches and mouse genetics to assess the role of TNF-receptor 1 and 2 in lesion-induced excitatory synaptic strengthening. Our experiments disclose that both TNF-receptors are involved in the regulation of denervation-induced synaptic plasticity. In line with this result TNF-receptor 1 and 2 mRNA-levels were upregulated after deafferentation *in vitro*. These findings implicate TNF-receptor signaling cascades in the regulation of homeostatic plasticity of denervated networks and suggest an important role for TNFα-signaling in the course of neurological diseases accompanied by deafferentation.

Homeostatic control of cortical excitability, connectivity and plasticity is considered essential for normal brain function^1^,^2^,^3^. Work from the past years has unraveled a wealth of information on compensatory mechanisms acting in the brain to keep the activity in neuronal networks within a physiological range^4^. Among the best studied mechanisms is homeostatic synaptic plasticity^5^,^6^,^7^,^8^, which adjusts synaptic strength to changes in network activity in a compensatory manner. Several studies have investigated the cellular and molecular mechanism of homeostatic synaptic plasticity in response to prolonged pharmacological inhibition of network activity with tetrodotoxin, inhibition of ionotropic glutamate receptors and/or blockade of voltage gated calcium channels^9^,^10^,^11^,^12^. Moreover, experimental evidence has been provided that homeostatic synaptic plasticity is induced in experimental models of synaptic deprivation *in vivo*^13^,^14^,^15^,^16^.

An important factor implied in the regulation of synaptic plasticity is the pro-inflammatory cytokine TNFα^17^,^18^,^19^,^20^,^21^,^22^,^23^, which acts via two canonical receptors: TNF-receptor 1 (TNFR1) and TNF-receptor 2 (TNFR2)^24^,^25^. TNFR1 predominantly binds soluble TNFα^26^,^27^ and is constitutively expressed on most cells of the body^28^. TNFR2 binds with high affinity to membrane-bound TNFα and is mainly expressed on cells of the immune system^25^,^26^,^28^. In the CNS both receptors are detected on neurons or glial cells^18^,^23^,^29^,^30^. While experimental evidence has been provided that TNFR-signaling affects synaptic plasticity^17^, the role of TNFR-signaling in the regulation of synaptic plasticity under pathological conditions remains not well understood.

In the present study we employed organotypic entorhino-hippocampal slice cultures^31^ as a tool to assess the role of TNFRs in *denervation-induced homeostatic synaptic plasticity*. Using entorhinal denervation *in vitro* (Fig. 1A,B), we recently showed that homeostatic synaptic strengthening of excitatory synapses occurs in denervated neuronal networks^9^,^32^,^33^,^34^. Moreover, we were able to demonstrate that TNFα maintains the compensatory increase in excitatory synaptic strength after denervation^9^. The results of the present study disclose that both TNF-receptors are involved in the regulation of denervation-induced homeostatic synaptic plasticity. In line with this finding an upregulation of TNFR1- and TNFR2-mRNA levels was observed in the denervated zone. Hence, both TNFR-signaling pathways seem to be recruited under pathological conditions to homeostatically adjust excitatory synaptic strength in denervated brain regions. These findings appear to be of considerable relevance for neurological diseases associated with increased TNFα levels, neuronal cell death and subsequent denervation of connected brain regions^24^,^35^,^36^.

## Results

### Denervation-induced homeostatic synaptic plasticity is not altered in slice cultures prepared from TNFR1-deficient mice

**Figure 1:**
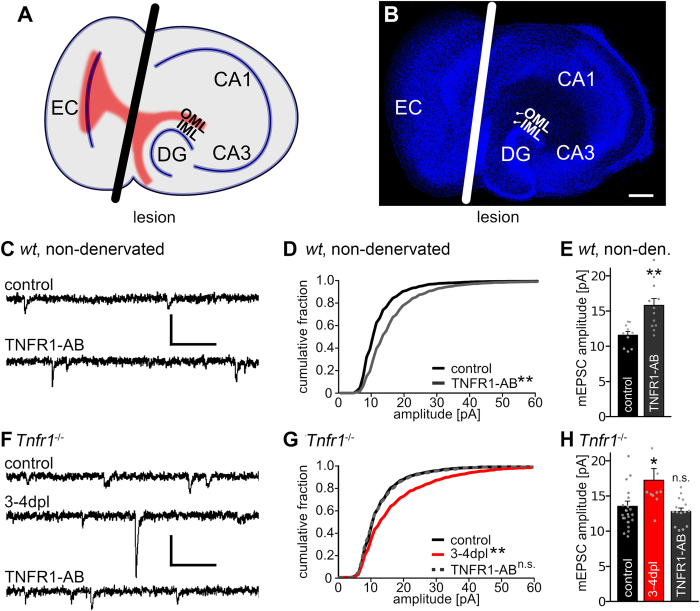
Activation of TNFα receptors leads to an increase in synaptic strength. (**A**) Schematic of an entorhino-hippocampal slice culture. Fibers from the entorhinal cortex are shown in red. The black line indicates the lesion (EC, entorhinal cortex; CA1, CA3, cornu ammonis 1, 3; DG, dentate gyrus; IML, OML, inner and outer molecular layer, respectively). (**B**) Example of an entorhino-hippocampal slice culture (blue: TO-PRO^®^ nuclear staining; white line: lesion site, scale bar: 200 μm). (**C**,**F**) Sample traces of whole-cell voltage-clamp recordings from dentate granule cells in non-denervated wild type slice cultures and in cultures prepared from TNFR1-deficient animals (*Tnfr1*^*−/−*^; dpl, days post lesion; scale bars: vertical, 20 pA; horizontal, 100 ms). (**D**,**E**,**G**,**H**) Cumulative distribution and mean values of mEPSC amplitudes. Treatment with TNFR1-AB (2 μg/ml) for 3 days increased mEPSC amplitudes significantly in *wild type* but not *Tnfr1*^*−/−*^ preparations. [wild type: controls: n = 10 cells; TNFR1-AB: n = 12 cells, from 4–6 cultures; *Tnfr1*^*−/−*^: controls: n = 16 cells; 3–4 dpl: n = 10 cells; TNFR1-AB: n = 15 cells, from 5–9 cultures; in D and G: Kolmogorov-Smirnov test; in E and H: Kruskal-Wallis-test followed by Dunn’s post hoc test; *indicates p < 0.05, **p < 0.01, n.s., not significant; in H one data point in the denervated group (3–4 dpl) is not shown: 30.1 pA].

Previous work has revealed that the activation of TNFR1 leads to an accumulation of GluA1-subunits on the surface of neurons within 24 h^17^ and could thus account for TNFα-mediated synaptic plasticity^9^,^30^,^37^. Therefore we hypothesized that TNFR1 could play a similar role in denervation-induced homeostatic synaptic plasticity (Fig. 1A,B). First, non-denervated wild type cultures were treated with a TNFR1-activating antibody (TNFR1-AB, 2 μg/ml; 3d)^17^, to confirm that the activation of TNFR1 can increase excitatory synaptic strength of mouse dentate granule cells in the slice culture preparations (Fig. 1C–E). Indeed, a significant increase in mean mEPSC amplitude was observed in TNFR1-AB treated non-denervated wild type cultures (Fig. 1D,E; mEPSC frequencies: control: 1.9 ± 0.3 Hz, TNFR1-AB: 4.7 ± 0.4 Hz, Kruskal-Wallis-test followed by Dunn’s post hoc test; p < 0.01).

In order to test for the role of TNFR1-signaling in denervation-induced homeostatic synaptic plasticity we employed slice cultures prepared from TNFR1-deficient mice (*Tnfr1*^*−/−*^; Fig. 1F–H). mEPSCs were recorded following denervation (at 3–4 dpl) and in age- and time-matched non-denervated control cultures. Unexpectedly, the cumulative distribution of mEPSC amplitudes was significantly changed, i.e., shifted rightward (Fig. 1G), and mean mEPSC amplitudes were significantly increased following denervation (Fig. 1H), while pharmacological activation of TNFR1 with TNFR1-AB (2 μg/ml; 3d) had no significant effect on mEPSC amplitudes in non-denervated TNFR1-deficient slice cultures (Fig. 1G,H; mEPSC frequencies *Tnfr1*^*−/−*^, control: 1.7 ± 0.2 Hz; 3–4 dpl: 2.8 ± 0.3 Hz, p < 0.05; TNFR1-AB: 1.4 ± 0.1 Hz, not significant; compared to control, Kruskal-Wallis-test followed by Dunn’s post hoc test). These experiments confirmed the lack of functional TNFR1 and suggested that signaling via the TNFR1 pathway is not essential for denervation-induced homeostatic synaptic strengthening to occur after entorhinal denervation *in vitro*.

### Denervation-induced homeostatic synaptic plasticity is observed in slice cultures prepared from TNFR2-deficient mice

**Figure 2:**
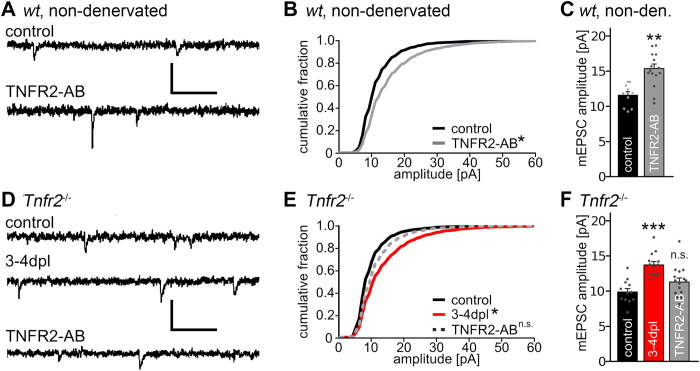
Denervation-induced homeostatic synaptic strengthening is observed in granule cells of TNFR1- or TNFR2-deficient slice cultures. (**A**,**D**) Sample traces of whole-cell voltage-clamp recordings from slice cultures prepared from wild type and TNFR2-deficient mice. Cultures were denervated and recorded after 3–4 days post lesion (dpl) or were not denervated and treated for 3 days with TNFR2 activating antibodies (TNFR2-AB; scale bars: vertical, 20 pA; horizontal, 100 ms). (**B**,**C**) Non-denervated wild type slice cultures were treated with TNFR2-AB for 3 days [controls: n = 10 cells (pooled data from Fig. 1E); TNFR2-AB: n = 15 cells; from 4–6 cultures; in C: Kolmogorov-Smirnov test; in B: Kruskal-Wallis-test followed by Dunn’s post hoc test; *indicates p < 0.05; **indicates p < 0.01]. (**E**,**F**) TNFR2-deficient granule cells (*Tnfr2*^*−/−*^) showed an increase in mEPSC amplitudes after denervation. In non-denervated cultures the activation of TNFR2 with the TNFR2-AB did not lead to an increase in mEPSC amplitudes, thus confirming the specificity of the activating antibody (controls: n = 12 cells; 3–4 dpl: n = 13 cells; TNFR2-AB: n = 15 cells; from 5–6 cultures; in E: Kolmogorov-Smirnov test; in F: Kruskal-Wallis-test followed by Dunn’s post hoc test; *indicates p < 0.05; ***indicates p < 0.001; n.s., not significant).

Since TNFα could act via TNFR2 in TNFR1-deficient preparations, we next tested for the role of TNFR2 in our experimental setting (Fig. 2). Similar to the experiments described above, changes in excitatory synaptic strength were first assessed after treatment of non-denervated wild type slice cultures with an activating antibody for TNFR2 (TNFR2-AB, 2 μg/ml; 3d). Indeed, in these experiments a significant increase in mEPSC amplitudes was observed (Fig. 2A–C; mEPSC frequencies: control: 1.9 ± 0.3 Hz, TNFR2-AB: 1.2 ± 0.2 Hz, not significant; Kruskal-Wallis-test followed by Dunn’s post hoc test).

Next, slice cultures prepared from TNFR2-deficient mice (*Tnfr2*^*−/−*^) were used to assess changes in mEPSCs following deafferentation (3–4 dpl). TNFR2-deficient granule cells showed a robust denervation-induced increase in excitatory synaptic strength (Fig. 2D–F; mEPSC frequencies *Tnfr2*^*−/−*^, control: 2.0 ± 0.3 Hz, 3–4 dpl: 4.0 ± 0.3 Hz, p < 0.001; TNFR2-AB: 2.9 ± 0.2 Hz, not significant; compared to control, Kruskal-Wallis-test followed by Dunn’s post hoc test), while TNFR2-AB had no significant effect on mEPSC amplitudes of dentate granule cells in non-denervated TNFR2-deficient slice cultures. These experiments confirmed the lack of functional TNFR2 and suggested that signaling via TNFR2 is not essential for denervation-induced homeostatic synaptic strengthening to occur after entorhinal denervation *in vitro*.

In the experiments outlined above, no significant difference between mean mEPSC amplitudes of the non-denervated TNFR1-deficient, TNFR2-deficient and wild type slice cultures was observed (c.f., Figs 1 and 2; wild type, control: 11.6 ± 0.5 pA; *Tnfr1*^*−/−*^, control: 13.6 ± 0.7 pA; *Tnfr2*^*−/−*^, control: 9.9 ± 0.5 pA; not significant, Kruskal-Wallis-test followed by Dunn’s post hoc test).

### Denervation-induced homeostatic synaptic plasticity is attenuated in slice cultures lacking TNFR1 and TNFR2

**Figure 3:**
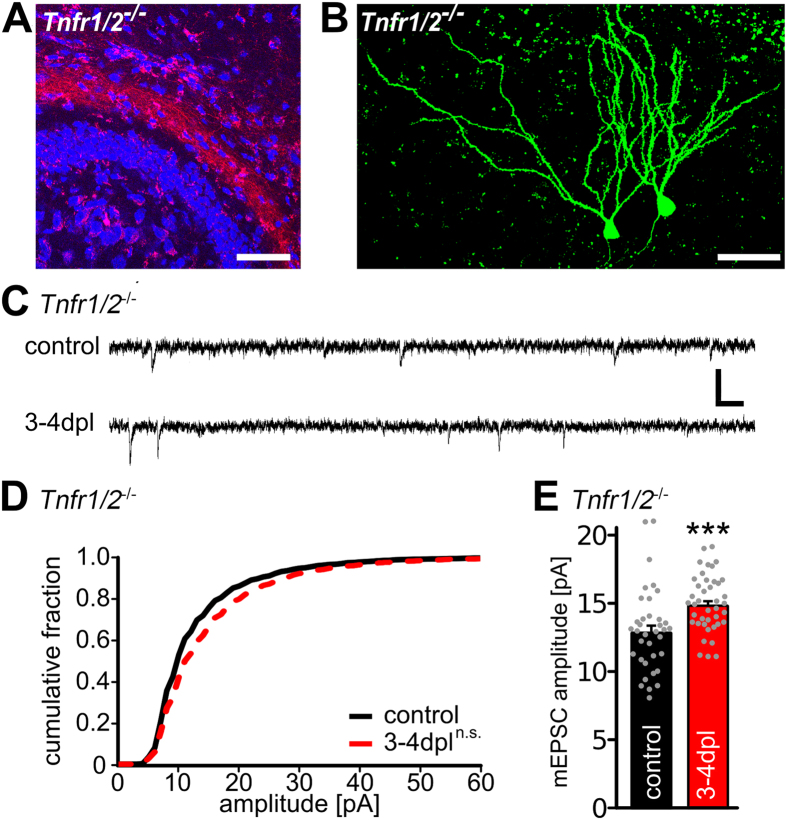
Granule cells deficient for both TNFRs show an impaired denervation-induced increase in synaptic strength. (**A**) Mini-Rubi tracing of entorhino-hippocampal axons in TNFR1/2-deficient slice cultures (*Tnfr1/2*^*−/−*^). Entorhinal fibers terminate in the outer molecular layer of the dentate gyrus (blue: TO-PRO^®^ nuclear staining, red: Mini-Rubi, scale bar: 100 μm). (**B**) Granule cells were patched with biocytin containing internal solution and stained with Alexa488-streptavidin (green) following the recording (scale bar: 50 μm). (**C**) Sample traces of whole-cell voltage-clamp recordings from granule cells of *Tnfr1/2*^*−/−*^ slice cultures. (**D**,**E**) Cumulative distributions and mean values of mEPSC amplitudes. Recordings were made from control and denervated (3–4 dpl) slice cultures (controls: n = 36 cells; 3–4 dpl: n = 41 cells, from 15–17 cultures in D: Kolmogorov-Smirnov test; in E: Mann-Whitney-test; ***p < 0.001; n.s., not significant). See also supplementary information.

In view of these results we speculated that the two TNFRs can compensate for each other with regard to denervation-induced synaptic strengthening. To test for this possibility denervation experiments were carried out in slice cultures prepared from mice lacking both TNFRs (Fig. 3A,B) and mEPSCs were recorded by 3–4 dpl (Fig. 3C–E). Under these conditions the increase of mEPSC amplitudes after denervation was less pronounced than in the single TNFR-deficient mice and the cumulative distributions of mEPSC amplitudes were not statistically different (Fig. 3D; see also supplementary information; Figure S1). Nevertheless, a significant difference between denervated and non-denervated cultures was observed with regard to mean mEPSC amplitudes values (Fig. 3E, mEPSC frequencies *Tnfr1/2*^*−/−*^, control: 1.6 ± 0.1 Hz, 3–4 dpl: 2.2 ± 0.2 Hz, not significant, Mann-Whitney-test). We concluded from these data that the denervation-induced increase in mEPSC amplitudes is attenuated but not completely abolished in granule cells lacking both TNFRs.

### Layer-specific upregulation of TNFR1- and TNFR2-mRNA denervation

**Figure 4:**
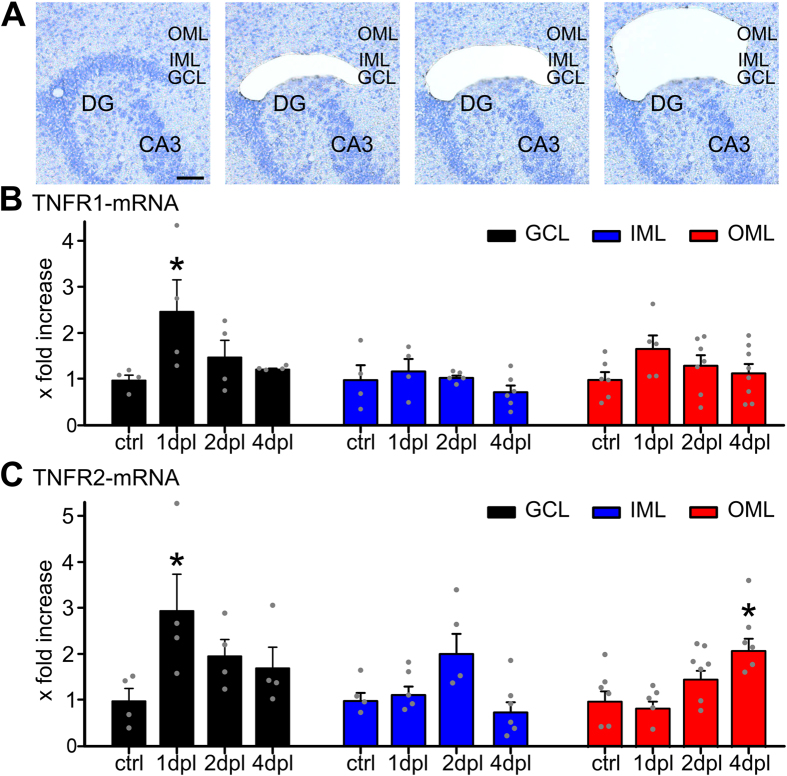
Laser capture microdissection combined with qPCR reveals changes in TNFR1- and TNFR2-mRNA levels following entorhinal denervation in vitro. (**A**) Laser capture microdissection (LMD) was employed to collect tissue from the granule cell layer (GCL), the inner molecular layer (IML), and the outer molecular layer (OML) of denervated cultures (at 1, 2, and 4 dpl) and non-denervated control cultures (scale bar: 50 μm). (**B**) TNFR1-mRNA levels were assessed in the isolated tissue using qPCR. An increase in TNFR1-mRNA was observed in the GCL at 1 dpl (n = 4–8 probes per group; Kruskal-Wallis-test followed by Dunn’s post hoc; *indicates p < 0.05; n.s., not significant). (**C**) TNFR2-mRNA levels were assessed in the isolated tissue using qPCR. An increase in TNFR2-mRNA was observed in the GCL at 1 dpl and in the OML at 4 dpl (n = 4–8 probes per group; Kruskal-Wallis-test followed by Dunn’s post hoc test; *indicates p < 0.05; n.s., not significant).

To further analyze the role of TNFRs in the process of synaptic strengthening after denervation and to test whether both receptors are regulated following entorhinal denervation *in vitro*, mRNA levels of TNFR1 and TNFR2 were determined in the cell and fiber layers of the dentate gyrus in wild type slice cultures (Fig. 4). Using laser capture microdissection, tissue was collected from the granule cell layer, i.e., the layer in which the cell bodies of granule cells are located, the non-denervated inner molecular layer and the denervated outer molecular layer, respectively (Fig. 4A). mRNA levels of TNFR1 and TNFR2 in these layers were analyzed at 1 dpl, 2 dpl and 4 dpl and compared to non-denervated controls using qPCR as previously described^9^,^34^. In these experiments an increase of TNFR1- and TNFR2-mRNA levels was observed in the granule cell layer at 1 dpl and a significant increase of TNFR2-mRNA levels in the outer molecular layer at 4 dpl (Fig. 4B,C). We concluded from these data that denervation-induced homeostatic synaptic plasticity is accompanied by an increase in TNFR1 and TNFR2, which corroborates our results on the involvement of both receptors in denervation-induced plasticity.

## Discussion

In this study the role of TNFRs in denervation-induced excitatory synaptic strengthening of dentate granule cells was determined using entorhino-hippocampal slice cultures. Our data suggest that signaling via either TNFR1 or TNFR2 is sufficient to induce synaptic strengthening and that the two TNFRs can, at least in part, compensate for each other with regard to homeostatic synaptic plasticity following entorhinal denervation *in vitro*.

Consistent with work published by other groups^17^,^38^ we could demonstrate that activation of TNFR1 leads to an increase in excitatory synaptic strength of non-denervated cultured dentate granule cells. This effect of TNFR1-AB was specific since treatment of TNFR1-deficient slice cultures did not show an increase in excitatory synaptic strength. An increase in mean mEPSC frequency was observed upon TNFR1-activation, which is consistent with the previously reported TNFα-mediated modulation of presynaptic properties by astrocytes^19^. In contrast to previous work^17^,^38^, however, we also observed an increase in synaptic strength following TNFR2 activation. Although TNFR2 activation had no effect on mEPSC frequencies in these experiments, the magnitude of the effect on mEPSC amplitudes was similar to the one seen after TNFR1-activation. This effect was specific for TNFR2, since it was abolished in TNFR2-deficient cultures treated with TNFR2-AB. Together, these observations indicate that signaling via either TNFR can induce synaptic strengthening in dentate granule cells of entorhino-hippocampal slice cultures, which is consistent with a partial overlap of TNFR1 and TNFR2 signaling pathways^24^,^25^. Whether TNFR1- and/or TNFR2-mediated strengthening of excitatory synapses in non-denervated control cultures resembles homeostatic or Hebbian-type of plasticity is not clear at present. It may also be important to mention in this context that glial cells of slice cultures return to their resting state within the first week after preparation^39^, suggesting that the effects of TNFR activation in our three-week old non-denervated control cultures are unlikely to be the result of a persisting neuroinflammatory response following the preparation of slice cultures. Nevertheless, TNFR-expression patterns and the precise TNFR-signaling pathways may differ between slice culture preparations and the mixed dissociated neuronal-glial cultures used by others^17^, which could explain some differences in the findings.

While the precise effects of TNFR-activating antibodies on distinct forms of plasticity warrant further investigation, the main question that we addressed in this study was the role of TNFRs in denervation-induced homeostatic synaptic strengthening following entorhinal denervation *in vitro*^33^. In previous work we have shown that entorhinal denervation results in homeostatic synaptic strengthening of surviving granule cell synapses, which functionally counteracts the denervation-induced reduction in spine numbers^33^,^40^. This form of homeostatic plasticity follows a characteristic time course with an initial induction phase of synaptic strengthening (1–2 dpl) followed by a maintenance phase (3–4 dpl). As we have previously reported, the maintenance phase but not the induction phase was found to be TNFα-dependent^9^. We now wondered whether this effect of TNFα is mediated by TNFR1, as suggested previously^17^,^30^,^41^. Accordingly, we used TNFR1-deficient mice and expected an impaired denervation-induced strengthening response in slice cultures generated from these mice. Surprisingly, we recorded a normal strengthening response from denervated TNFR1-deficient granule cells, suggesting that TNFα does not mediate its effect exclusively via TNFR1. A similar result was obtained using TNFR2-deficient preparations. Together with the observation that in non-denervated control cultures activation of either TNFR1 or TNFR2 can induce synaptic strengthening, these observations suggested that the two TNFRs compensate for each other in denervation-induced synaptic strengthening.

Because of the above results we investigated mice deficient for both TNFRs. In these preparations an attenuated synaptic strengthening response was observed. Although an increase in average mEPSC amplitudes after 3–4 dpl was still detectable, the cumulative distribution of mEPSC amplitudes did not show changes characteristic for a homeostatic increase in excitatory synaptic strength — as seen in pharmacologically induced synaptic scaling^10^ or following entorhinal denervation *in vitro*^9^,^33^. We conclude from this impaired response seen in TNFR1/2-deficient preparations that TNFR-signaling plays a role in denervation-induced synaptic strengthening.

Why do TNFR1/2 mutants still show an increase in average mEPSC amplitudes, whereas the strengthening effect is absent in mice lacking the ligand, i.e., TNFα^9^? One possibility are the known limitations of models of constitutive gene deficiency. For example, developmental disturbances could have influenced our experiments. Although we have shown that the entorhino-hippocampal projection develops in slice cultures of these mice and that basal synaptic transmission is comparable, we cannot fully exclude that unknown developmental impairments might have affected our results. This appears to be a possibility since TNFR1/2-deficient mice exhibit increased neuronal degeneration and reduced activity of microglia in response to brain injury^21^. Also, cultured neurons that are not expressing TNFR1 have significantly less GluA1 clusters associated with PSD95^30^. This reduction in GluA1 should, however, lead to an altered baseline synaptic transmission in non-denervated granule cells. To control for this possibility we compared baseline mEPSC amplitudes of TNFR1- and TNFR2-deficient mice with wild type granule cells, but did not find a significant difference. Thus, although we cannot fully exclude this possibility, we also have no positive evidence for developmental disturbances which could explain our findings.

Another explanation for our results is the possibility that after denervation TNFα may not only exert its effects via the two known canonical receptors. Since several other pathways have been described which could lead to an increase in synaptic strength^5^,^6^,^7^,^8^, yet unidentified non-canonical pathways could contribute to TNFα signaling following denervation. It is also conceivable that other, i.e., TNFα-independent compensatory mechanisms could be recruited under conditions of TNFR1/2-deficiency, including compensation by other cytokines (e.g., IL-1β^17^), other homeostatic synaptic mechanisms or for example compensatory changes in active and passive properties of denervated dentate granule cells. Further investigations will be needed to address these questions and to test for the possibility of a cross-talk between various signaling pathways and distinct homeostatic mechanisms. In this context it will be also important to determine the role of TNFR-signaling in denervation-induced structural reorganisation^42^, i.e., changes in spine turnover^40^, dendritic plasticity^43^,^44^ and/or collateral axonal sprouting^45^. It is interesting to speculate that TNFa could orchestrate various aspects of denervation-induced functional and structural plasticity by acting on TNFRs in distinct neural compartments.

Regardless of these considerations, the LMD-qPCR experiments disclosed that mRNA levels of both TNFRs increase in the granule cell layer of wild type cultures following entorhinal denervation *in vitro*. These changes could make denervated granule cells (and/or glial cells; note TNFR2-mRNA increase in the OML at 4 dpl; c.f.,^9^) more sensitive to TNFα-signaling. The results are consistent with a previous study which showed that granule cells can express both TNFR in response to toxic stimuli^23^ and with an *in vivo* study which reported an upregulation of TNFR1-mRNA after entorhinal cortex lesion^41^. While the neural compartments in which TNFα-signaling via TNFR1 and TNFR2 takes place and the precise contribution of each TNFR to denervation-induced network remodeling need to be determined, the presence and upregulation of both TNFRs in granule cells supports our major conclusion that signaling via either TNFR can mediate the synaptic strengthening response after denervation. Hence, coordinated changes in TNFα-expression^9^ and TNFRs (this study) may account for distinct phases of denervation-induced homeostatic plasticity and may contribute to the dose-dependent and diverse effects of TNFα reported under different experimental conditions.

## Materials and methods

### Preparation of slice cultures

Experimental procedures were performed in agreement with the German law on the use of laboratory animals and approved by the animal welfare officer of Goethe-University Frankfurt (Faculty of Medicine). Entorhino-hippocampal slice cultures were prepared at postnatal day 4–5 as previously described (e.g.^9^). C57BL/6J, TNFR1-deficient, TNFR2-deficient and TNFR1/2-deficient mice^46^ and wild type littermates of either sex were used. Transgenic animals were obtained from Jackson Laboratories, USA. Slice cultures were allowed to mature for ≥18 days *in vitro* (div) in humidified atmosphere with 5% CO_2_ at 35 °C before experimental assessment.

### Entorhinal cortex lesion

Slice cultures (18–25 div) were transected using a sterile scalpel blade (Fig. 1A,B; e.g.^33^). To ensure complete and permanent separation of the entorhinal cortex from the hippocampus the entorhinal cortex was removed from the culturing dish.

### Perforant path tracing

Anterograde tracing of the entorhino-hippocampal pathway with biotinylated and rhodamine conjugated dextran amine (Mini-Rubi, Molecular Probes, USA, c.f., Fig. 3A) was performed as described previously^9^.

### Whole-cell patch-clamp recordings

Whole-cell voltage-clamp recordings and post hoc identification of recorded neurons (Fig. 3B) were carried out as previously described^9^. Age- and time-matched non-denervated cultures prepared from the same animal or littermate animals served as controls. Non-denervated control (or untreated) cultures were recorded alternating with the recordings of denervated and/or treated cultures (c.f.,^33^). All recordings were performed at 35 °C in artificial cerebrospinal fluid (ACSF; 126 mM NaCl, 2.5 mM KCl, 26 mM NaHCO_3_, 1.25 mM NaH_2_PO_4_, 2 mM CaCl_2_, 2 mM MgCl_2_, and 10 mM glucose) saturated with 95% O2 / 5% CO2. For miniature excitatory postsynaptic current (mEPSC)-recordings 10 μM (2R)-amino-5-phosphonovaleric acid (D-APV) and 0.5 μM tetrodotoxin (TTX) were added to ACSF. Patch-pipettes (4–5 MΩ resistance) contained 126 mM K-gluconate, 4 mM KCl, 4 mM ATP-Mg, 0.3 mM GTP-Na_2_, 10 mM PO-Creatine, 10 mM HEPES and 0.3% Biocytin (pH = 7.25 with KOH, 280 mOsm with sucrose). Recordings were carried out with a Multiclamp 700B amplifier (Molecular Devices) at a holding potential of −70 mV. Signals were low-pass filtered at 6 kHz (Bessel filter) and sampled at 10 kHz with pClamp software (Molecular Devices). Pipette and cell capacitance transients were compensated. Series resistance was not compensated and monitored in 2 min intervals. Recordings were discarded if the series resistance and leak current changed significantly during recording and/or reached ≥30 MΩ or ≥50 pA, respectively.

### Drug treatments

Non-denervated slice cultures were treated with activating antibodies for TNFR1 or TNFR2 (TNFR1-AB and TNFR2-AB, 2 μg/ml; AF-426-PB, AF-425-PB, R&D systems, USA) for 3d.

### Laser capture microdissection (LMD) of re-sliced cultures

Slice cultures were washed with phosphate buffered saline (PBS; 0.1 M, pH 7.4), shock frozen at −80 °C in tissue freezing medium (Leica Microsystems, Germany), re-sliced into 10 μm thick slices on a cryostat (Leica CM 3050 S) and mounted on PET foil metal frames (Leica, Germany) as described previously^9^. Re-sliced cultures were fixed in ice-cold acetone for 1 min and incubated with 0.1% toluidine blue (Merck, Germany) at room temperature for 1 min, before rinsing in ultrapure water (DNase/RNase free, Invitrogen, USA) and 70% ethanol. PET foil metal frames were mounted on a Leica DM 6000B LMD system (Leica Microsystems, Germany) with the section facing downward. After adjusting intensity, aperture, and cutting velocity, the pulsed ultraviolet laser beam was carefully directed along the borders of the respective hippocampal layers of interest using a 20× objective lens (Leica Laser Micro dissection, Software Version 7.4.1.4853). Tissue samples from the OML, IML and GCL of the suprapyramidal blade of the dentate gyrus were collected (Fig. 4A). Care was taken to isolate areas of approximate same size (~300 × 10^3^ μm) from each slice (for IML for each probe 2 cultures were pooled to get approximately the same size of tissue per group). Microdissected tissue was transferred by gravity into micro centrifuge tube caps placed underneath the sections, filled with 50 μl guanidine isothiocyanate (GITC)-containing buffer (RLT Buffer, RNeasy Mini Kit, Qiagen, Germany) with 1% ß-mercaptoethanol (AppliChem GmbH; Germany). Tissue collection was visually verified. All samples were frozen and stored at −80 °C.

### Isolating RNA and qRT-PCR

RNA was isolated using the RNeasy® MicroPlus Kit (Qiagen, Germany; all kits and assays were used according to the manufacturer’s instructions). Purified RNA (RNA integrity number, RIN: 7.8 ± 0.1; Agilent 2100 Bioanalyzer system and Agilent RNA 6000 Pico Kit; Agilent Technologies, Germany) was transcribed into cDNA with the High Capacity cDNA Reverse Transcription Kit (Applied Biosystems, USA). The cDNA was amplified using the TaqMan®PreAmp Master Mix Kit (Applied Biosystems, USA, TaqMan Gene Expression(TM)-Assay GAPDH: 4352932E; TNFR1: Mm00441875_m1; TNFR2: Mm00441889_m1) with a standard amplification protocol (14 cycles: 95 °C for 15 sec; 60 °C for 4 min). Amplified cDNAs were diluted 1:20 in ultrapure water and subjected to qPCR (StepOnePlus, Applied Biosystems, USA) using a standard amplification program (1 cycle of 50 °C for 2 min, 1 cycle of 95 °C for 10 min, 40 cycles of 95 °C for 15 sec and 60 °C for 60 sec; cut off at 31 cycles; average CT-value for GAPDH: 16.8 ± 0.5 cycles, for TNFR1: 23.6 ± 0.9 cycles, for TNFR2: 27.4 ± 1.4 cycles).

### Immunohistochemistry

Cultures were fixed in a solution of 4% (w/v) PFA and 4% (w/v) sucrose in PBS for 15 min. Fixed slice cultures were thoroughly washed and stained with Alexa488 or Alexa568 coupled streptavidin antibodies (1:200) in PBS with 10% (v/v) normal horse serum and 0.1% (v/v) Triton X-100 for 2–3 hours. Cultures were incubated with TO-PRO^®^-3 IODID (1:5000 in PBS for 3 min; Invitrogen, USA) to obtain nuclear staining.

### Microscopy

Traced entorhino-hippocampal fibers and post-hoc identified recorded neurons were visualized using a Nikon Eclipse C1si laser-scanning microscope equipped with a 40× oil-immersion (NA 1.3, Nikon) and 60× oil-immersion (NA 1.4, Nikon) objective lens. Images were acquired with EZ-C1 software 3.6 for Nikon C1 confocal and stored as .tif or .ics files.

### Quantification and statistics

Electrophysiological data were analyzed using pClamp 10.2 (Axon Instruments, USA) and MiniAnalysis (Synaptosoft, USA) software. All events were visually inspected and detected by an investigator blind to experimental condition. 250–350 events were analyzed per recorded neuron. No significant differences between age- and time-matched non-denervated cultures were observed, c.f.^33^. Similarly no differences between 3 dpl vs. 4 dpl were observed. Therefore these data (control, 3–4 dpl) were pooled.

qPCR-data were analyzed as described by Pfaffl^47^. GAPDH served as reference gene in this analysis. The qPCR assay efficiency was calculated with the StepOnePlus software (Applied Biosystems, USA) based on a dilution series of 5 samples for each assay. Data of age- and time-matched non-denervated control cultures were pooled.

Statistical comparisons were made using non-parametric tests, since normal distribution of data could not be assured: Mann-Whitney-test (for comparing two groups) or Kruskal-Wallis-test followed by Dunn’s post hoc analysis (if more than two groups were compared). Statistical analysis of cumulative mEPSC amplitude distributions were made with Kolmogorov-Smirnov test. All statistics were calculated using GraphPad Prism 6 (GraphPad software, USA). P-values of less than 0.05 were considered a significant difference. All values are expressed as mean ± standard error of the mean (SEM; absolute values and medians, i.e., box-whisker-plots given in the supplementary information; Figures S2-S4).

### Digital Illustrations

Confocal image stacks were exported as 2D-projections and stored as TIF files. Figures were prepared using ImageJ^48^ and Inkscape (download: www.inkscape.org). Image brightness and contrast were adjusted.

## Additional Information

**How to cite this article**: Becker, D. *et al.* Tumor necrosis factor (TNF)-receptor 1 and 2 mediate homeostatic synaptic plasticity of denervated mouse dentate granule cells. *Sci. Rep.* **5**, 12726; doi: 10.1038/srep12726 (2015).

## Supplementary Material

Supplementary Information
