# Supplementary material for: Tumor necrosis factor (TNF)-receptor 1 and 2 mediate homeostatic synaptic plasticity of denervated mouse dentate granule cells
Source: Sci Rep. 2015 Aug 6;5:12726. doi: 10.1038/srep12726 (PMC4526848; doi:10.1038/srep12726)
Supplement: Supplementary Information [file srep12726-s1.pdf]

SUPPLEMENTARY INFORMATION:

Tumor necrosis factor (TNF)-receptor 1 and 2 mediate homeostatic synaptic plasticity of denervated mouse dentate granule cells by Denise Becker, Thomas Deller, Andreas Vlachos

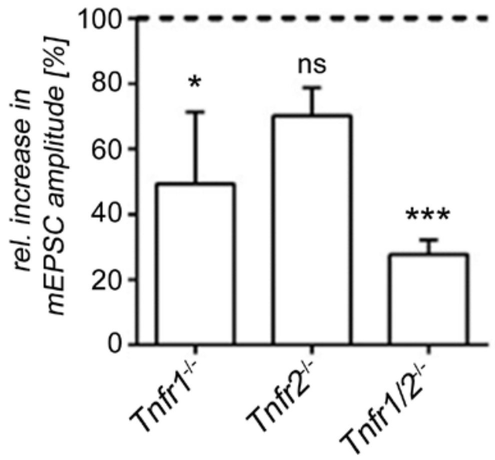

Figure S1: Relative increase in mEPSC amplitudes of denervated dentate granule cells from TNFR deficient preparations (3 - 4 days post lesion; dpl) compared/normalized to the denervation-induced homeostatic synaptic strengthening seen in wild type preparations at 3 - 4 dpl (wild type data taken from Becker et al., 2013; wildtype: 100 ±15 %; *Tnfr1*$^{-/-}$: 49 ±21 %; *Tnfr2*$^{-/-}$: 70 ±8 %; *Tnfr1/2*$^{-/-}$: 27 ±4 %, n = 10-41 cells; at least 4 cultures per group; Kruskal-Wallis-test followed by Dunn's post-hoc-test; * $p < 0.05$; *** $p < 0.001$; ns, not significant).

Reference:
Becker, D., Zahn, N., Deller, T. & Vlachos, A. Tumor necrosis factor alpha maintains denervation-induced homeostatic synaptic plasticity of mouse dentate granule cells. *Front Cell Neurosci* **7**, 257, doi:10.3389/fncel.2013.00257 (2013).

Figure S2: Wildtype slice cultures treated with TNFR-activating antibodies for 3 days

	mEPSC amplitude [pA]			mEPSC frequency [Hz]		
**GROUP**	**control**	**TNFR1-AB treated**	**TNFR2-AB treated**	**control**	**TNFR1-AB treated**	**TNFR2-AB treated**
**wild type**	11.6 ± 0.5 (10)	15.6 ± 1.0 (12); **	15.2 ± 0.6 (15); **	1.9 ± 0.3 (10)	4.7 ± 0.4 (12); **	1.2 ± 0.2 (15); [ns]

All values expressed as mean ± standard error of the mean; number of neurons recorded per group indicated in brackets: Kruskal-Wallis-test followed by Dunn's post-hoc correction; ** $p < 0.01$. ns, not significant

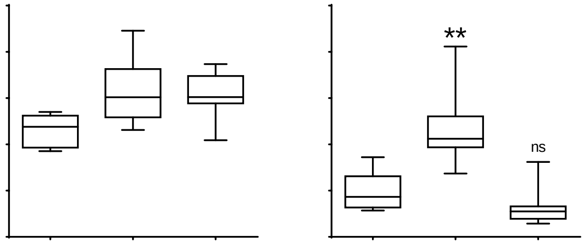

Box-Whisker-Plot of data from Table S1. ** $p < 0.01$; ns, not significant.

Figure S3: Slice cultures prepared from TNFR-deficient mice

	mEPSC amplitude [pA]			mEPSC frequency [Hz]		
**GROUP**	**control**	**3 - 4 days post lesion**	**TNFR-AB treated**	**control**	**3 - 4 days post lesion**	**TNFR-AB treated**
Tnfr1$^{-/-}$	13.6 ± 0.7 (16)	17.3 ± 1.6 (10); *	12.9 ± 0.5 (15); ^{ns}	1.7 ± 0.2 (16)	2.8 ± 0.3 (10); *	1.4 ± 0.1 (15); ^{ns}
Tnfr2$^{-/-}$	9.9 ± 0.5 (12)	13.8 ± 0.5 (13); ***	12.3 ± 0.6 (15); ^{ns}	2.0 ± 0.3 (12)	4.0 ± 0.3 (13);***	2.9 ± 0.2 (15); ^{ns}
Tnfr1/2$^{-/-}$	13.0 ± 0.5 (36)	15.0 ± 0.3 (41);***	-----------	1.6 ± 0.1 (36)	2.2 ± 0.2 (41);^{ns}	-----------

Tnfr1$^{-/-}$ treated with TNFR1-AB for 3d; *Tnfr2*$^{-/-}$ treated with TNFR2-AB for 3d; all values expressed as mean ± standard error of the mean; number of neurons recorded per group indicated in brackets: Kruskal-Wallis-test followed by Dunn's post-hoc correction for *Tnfr1*$^{-/-}$ and *Tnfr2*$^{-/-}$; Mann-Whitney-test for *Tnfr1/2*$^{-/-}$; * $p < 0.05$; *** $p < 0.001$. ns, not significant

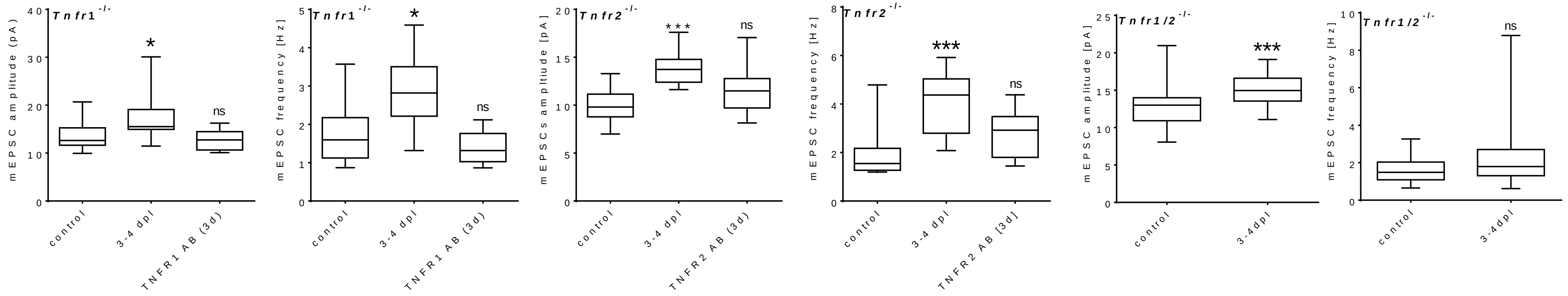

Box-Whisker-Plot of data from Table S2. days post lesion (dpl). * $p < 0.05$; *** $p < 0.001$; ns, not significant.

Figure S4: qPCR analysis of TNFR-mRNA levels

	TNFR1-mRNA (x fold change)				TNFR2-mRNA (x fold change)			
**GROUP**	**control**	**1 dpl**	**2 dpl**	**4 dpl**	**control**	**1 dpl**	**2 dpl**	**4 dpl**
**GCL**	1.0 ± 0.1 (4)	2.5 ± 0.7 (4); *	1.5 ± 0.4 (4); ns	1.2 ± 0.02 (4); ns	1.0 ± 0.3 (4)	3.0 ± 0.8 (4); *	2.0 ± 0.4 (4); ns	1.7 ± 0.5 (4); ns
**IML**	1.0 ± 0.3 (4)	1.2 ± 0.3 (4); ns	1.0 ± 0.1 (5); ns	0.7 ± 0.1 (6); ns	1.0 ± 0.2 (4)	1.1 ± 0.2 (5); ns	2.0 ± 0.4 (4); ns	0.8 ± 0.2 (6); ns
**OML**	1.0 ± 0.2 (6)	1.7 ± 0.3 (5); ns	1.3 ± 0.2 (7); ns	1.1 ± 0.2 (8); ns	1.0 ± 0.2 (6)	0.9 ± 0.1 (5); ns	1.5 ± 0.2 (7); ns	2.1 ± 0.3 (6); *

All values expressed as mean ± standard error of the mean; number of probes per group indicated in brackets: Kruskal-Wallis-test followed by Dunn's post-hoc correction; * $p < 0.05$; *** $p < 0.001$. ns, not significant

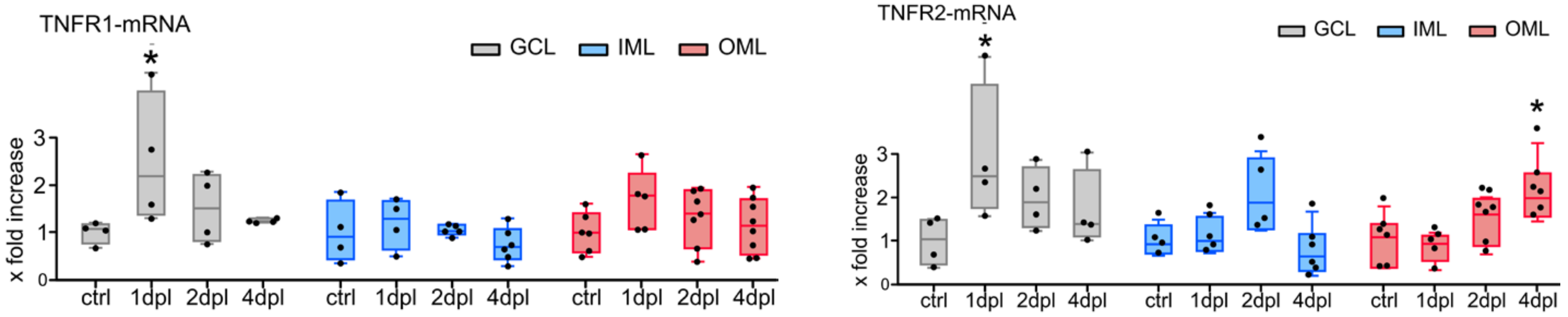

Box-Whisker-Plot of data shown in Table S3. Days post lesion (dpl). * $p < 0.05$.
